# Much or Little Water in Vulcanizing

**Published:** 1886-10

**Authors:** George B. Snow


ARTICLE IV.

### "MUCH OR LITTLE WATER IN VULCANIZING?"

I noticed recently in one of our leading dental offices that in vulcanixing a case the boiler was filled nearly to the brim with water. If those who still follow that method would put but a half-dozen spoonfuls in a steam-tight boiler, even placing something under the flask to keep it fiom the water, they may discover three advantages in the device; a tougher plate; absence of much disagreeable odor, and models and investments disintegrated. Give a little longer time for vulcanizing by this method. Those who offer new machines for accomplishing this purpose may not thank me for this suggestion.—*Dr. F. W. Williamson, Redwing, Minn., in Items of Interest.*

The above clipping gives advice which is right in one sense, and wrong in another. A series of recent experiments not yet fully completed, seems at this time to raise a serious doubt as to whether a piece of rubber vulcanized in steam and above water can be distinguished by its texture from another piece of the same rubber, properly vulcanized under water. The caution extended to "give a little longer time for vulcanizing," gives the clue to the superior qualities, if any there be, to the steam-vulcanized product. The mixture of air and steam, usually included in a "steam-tight" boiler, is not so good a conductor of heat as is pure steam. The flask will not be as hot by several degrees when resting in the steam-space as when placed under water, and it is undeniably the fact that a low temperature and long time produces the best results in vulcanizing.

Hence, if two flasks are placed in the same vulcanizer, one in the water and one in the steam-space, there will be quite a difference in the quality of the rubber vulcanized in them, both being subjected to the same time, and apparently the same heat.

Some of the first dental vulcanizers made, dating very early in the sixties, consisted of a boiler with a separate vulcanizing chamber above it, the flasks being thus placed in an atmosphere of steam, all condensation at once descending to the boiler. It was deemed a long step in advance when it was found that a single-chamber vulcanizer would answer all purposes, the vulcanization being done as well under water as in steam.

It is probable that the practice of covering the flasks with water arose from its being found that there was less difference in the hardness of the rubber in the different flasks when this was done. It is a well-known fact that such a difference exists, and is quite noticeable when the contents of the upper and lower flasks are compared, when three flasks are put in the vulcanizer, one above the other, even when all are covered by water. The reason for this difference is, that water is a poor conductor of heat, its temperature being equalized mainly by circulation or "convection." The space between the flasks and the walls of the vulcanizer being narrow, circulation of the water is necessarily obstructed to some extent, and the lower flask, receiving the heat directly as it is transmitted through the bottom of the vulcanizer, becomes a few degrees hotter than the upper one.

Now, if these three flasks were placed in the vulcanizer with "a few spoonsfuls of water," this difference of temperature will be no less, but rather greater, if they were in an atmosphere of mixed air and steam. If precautions are taken to expel all the air, the temperature will be uniform throughout the vulcanizer, and the vulcanizing uniform in all the flasks. As vulcanizing is usually done, with more or less air included in the vulcanizer, the actual temperature of the water may be as much as twenty degrees higher than the indication of the thermometer, which only gives the temperature of the vulcanizer cover upon which it is fixed. This is shown by the fact that a steam-gauge and a thermometer, mounted upon the same vulcanizer, cannot be made to agree in their indications unless all air is expelled from the vulcanizer.

A very important factor in doing good vulcanizing, is an even temperature; any sudden variations are sure to be shown in the quality of the work. Spongy rubber is sometimes caused by a sudden fall in temperature and pressure. This is one of the principal reasons for the uniform excellence of the work done by the gas-regulator, as this device automatically keeps the temperature at the desired point when it has once been attained.

There is a good reason for not filling the vulcanizer too full of water, aside from any effect on the vulcanizing process. If there is not sufficient steam room above the water, its expansion by heat will cause it to blow out the safety disc before the vulcanizing point is reached; or, in an extreme case, if there is no relief, the vulcanizer may burst under the strain. This is an entirely different thing from steam pressure; there will be no explosion, but the vulcanizer will give way and relieve the strain.

Water is not perceptibly elastic, and its expansion by heat in a chamber which it perfectly fills will give rise to a tremendous pressure, which, however, will be wholly relieved by a slight yielding of the walls of the chamber, or a small escape. A Whitney vulcanizer was once returned to us with the sides of its pot stretched to a quarter of an inch greater than the original diameter. On inquiry it was found that it was the effect of several vulcanizations, the vulcanizer each time being filled "brim full" of water. This was in the day of fusible safety-plugs, which did not give way to pressure, but only when the temperature rose above the vulcanizing point.

In another case, trouble was experienced with a Hayes boiler, the discs constantly blowing out. The dentist finally put on three at once, and was rewarded for his trouble by a rupture of the vulcanizer bottom. The cause of all the trouble was found to be, on injury, "filling the vulcanizer full of water."

The desire for quickening the vulcanizing process has led to the use of too high a temperature; hence the numerous complaints of brittle plates and spongy rubber.

Superior results can be attained with any good vulcanizer by a low heat, say 300°, the time being lengthened accordingly. The plate may be placed under water or in steam; but to secure any certainty that the thermometer will indicate the true temperature of the interior of the vulcanizer, all air must be expelled from it by blowing off steam when it is first forming. Then the rubber will not be over-heated, and all the work will be evenly done.—GEO. B. SNOW *in Dental Advertiser.*

